# P98 - Gluten allergy

**DOI:** 10.1186/2045-7022-4-S1-P153

**Published:** 2014-02-28

**Authors:** Estefânia Barrosa Maia, Loureiro Carla Chaves, Sónia Lemos, José Antonio Pinheiro

**Affiliations:** 1 Hospital Pediatrico, Centro Hospitalar e Universitário de Coimbra, Coimbra, Portugal

## Background

Gluten is a mix of cereals proteins (glutenins and gliadins) and can cause two distinct immunological diseases: celiac disease and IgE-mediated gluten allergy (GA). There are few cases of GA reported and little is known about its natural history.

## Methods

Description of GA in children followed at ours Pediatric Allergy Clinic: clinical manifestations, laboratory data, comorbidities and follow-up.

## Results

Nine children with GA are followed at our Pediatric Allergy Clinic, with ages ranging from 1-14 years and 8 are boys. Early presentation occurred between 5 and 9 months of age: 6 with cutaneous manifestations, 2 with gastrointestinal manifestations and 1 with anaphylaxis. The route of sensitization was cutaneous (with oat cream) and by food ingestion. Serum specific IgE to gluten ranged from 0,73 to 100KUI/L. Four children had specific IgE to other cereals without gluten (corn and rice). All children exhibited other atopic diseases. Eight children had multiple food allergies (egg, milk or fish). Four children developed respiratory allergy (asthma and rhinitis) and 2 of them had specific IgE to cereals pollens. All children adopted a gluten free diet and have adrenaline self-injectors. Two patients suffered anaphylaxis episodes after the diagnosis and 3 children resolve their GA (1, 2 and 6 years).

## Conclusions

GA is a rare disease, and can be life-threatening. Clinical presentation occurs in the first year of life and cutaneous manifestations are the most common. This allergy may resolve during childhood or become persistent. Atopic dermatitis can be an early manifestation of disease and the route of sensitization can be cutaneous. Exacerbation of atopic dermatitis with oat creams may alert to this diagnosis. Multiple food allergy is frequent in children with GA.

